# Lipid-Derived Cardiometabolic Indices in Normouricemic and Hyperuricemic Adults: A Retrospective Cross-Sectional Association Study

**DOI:** 10.3390/healthcare13233151

**Published:** 2025-12-03

**Authors:** Yazeed Alshuweishi, Salihah H. Khobrani, Muath Alsaidan, Tahani M. Alharthi, Mohannad G. Abdelgader, Abdulaziz M. Almuqrin

**Affiliations:** 1 Chair of Medical and Molecular Genetics Research, Department of Clinical Laboratory Sciences, College of Applied Medical Sciences, King Saud University, Riyadh 12372, Saudi Arabia; aalmuqrin@ksu.edu.sa; 2 Central Research Laboratory, King Saud University, Riyadh 12372, Saudi Arabia; 445819188@kku.edu.sa; 3 Department of Family and Community Medicine, College of Medicine, King Saud University, Riyadh 12372, Saudi Arabia; 4 Department of Clinical Nutrition, King Khalid University Hospital (KKUH), Riyadh 12372, Saudi Arabia; talharthi1@ksu.edu.sa; 5 Department of Microbiology and Clinical Immunology, Hakeem Medical Laboratories, Al Khobar 34422, Saudi Arabia; mohannad.abdelgader@hakeemlabs.com

**Keywords:** hyperuricemia, triglyceride–glucose index (TyG), remnant cholesterol (RC), non-HDL cholesterol, atherogenic index of plasma (AIP), Castelli’s risk indices

## Abstract

**Introduction:** Hyperuricemia is increasingly recognized as a metabolic disorder linked to dyslipidemia, insulin resistance, and vascular complications. In Saudi Arabia, the prevalence of hyperuricemia is rising with obesity and diabetes, yet its relationship with lipid-derived cardiometabolic indices remained understudied. This study aimed to examine the associations between uricemia status and lipid-derived cardiometabolic indices in a large adult cohort. **Methods:** This retrospective cross-sectional study analyzed data from 7652 adults, including 5385 normouricemic (NU) and 2267 hyperuricemic (HU). Key cardiometabolic indices, including the triglyceride-glucose index (TyG), non-high-density lipoprotein cholesterol (non-HDL-C), remnant cholesterol (RC), atherogenic index of plasma (AIP), and Castelli risk indices I and II (CRI-I, CRI-II), were calculated. Associations were evaluated treating HU as the exposure and the lipid-derived cardiometabolic indices as the outcomes. Multivariable regression analyses, receiver operating characteristic (ROC) curves, and prevalence-based association estimates were used to assess these relationships. **Results:** HU individuals exhibited significantly higher TG along with lowered HDL-C. Median TyG (4.61), AIP (0.38), non-HDL-C (147 mg/dL), RC (18 mg/dL), CRI-I (4.30), and CRI-II (2.85) were higher in the HU group compared to NU group, with non-HDL-C and CRI-I falling within the abnormal range, AIP in the high-risk range, and TyG and CRI-II at borderline levels. Across the separately adjusted models, hyperuricemia showed consistent positive associations with RC, AIP, CRI-I, and CRI-II, whereas associations with TyG and non-HDL-C diminished after adjustment for renal or liver markers. ROC analysis demonstrated modest discriminatory ability of uric acid for elevated indices, with AIP (AUC = 0.641) and CRI-I (AUC = 0.640) exhibiting the highest performance. The prevalence of elevated indices was substantially higher in HU, particularly for CRI-II (44.0% vs. 25.9%) and CRI-I (28.2% vs. 13.7%). **Conclusions:** These findings highlight associations between HU and lipid-derived cardiometabolic indices, but further longitudinal research is required to determine whether HU has a clinical predictive value in cardiovascular risk assessment.

## 1. Introduction

Hyperuricemia (HU), increasingly prevalent worldwide and recognized as an emerging metabolic and cardiovascular risk factor, is defined as serum uric acid concentrations exceeding the established sex- and age-specific reference intervals, typically 3.5–7.0 mg/dL in men and postmenopausal women and 2.6–5.7 mg/dL in premenopausal women, while levels above 6.8 mg/dL represents the biochemical solubility limit of monosodium urate, above which the likelihood of urate supersaturation and crystallization increases substantially [1]. In Saudi Arabia, recent evidence suggests a growing burden of HU that parallels the rise in obesity, diabetes, and other cardiometabolic conditions [2,3]. In a large national sample, the prevalence of HU was reported at 17.3%, with men demonstrating a higher prevalence than women (20.3% vs. 15.1%) [4]. Earlier estimates, however, were lower; for example, Al-Arfaj and colleagues observed an 8.4% prevalence among adults in Riyadh, reflecting both temporal increases and methodological differences [5]. These findings indicate that HU is becoming more common in Saudi Arabia, with recent rates aligning more closely with those observed in Western populations. Recent epidemiological studies indicate that the prevalence of HU is approximately 20–25% in the United States based on NHANES data [6,7], while in China, it ranges from 13–20%, depending on region and population characteristics [8,9].The rising prevalence in Saudi Arabia is of particular concern given the Kingdom’s high rates of metabolic syndrome, obesity, and dietary patterns rich in red meat, sugary beverages, and refined carbohydrates, all of which are known contributors to elevated uric acid [10,11].

Although traditionally associated with gout and urate crystal arthropathies, HU has increasingly been recognized as a systemic metabolic disturbance with far-reaching consequences. Elevated uric acid can lead to renal complications such as nephrolithiasis, uric acid nephropathy, and progression of chronic kidney disease [12,13,14]. It is also implicated in the pathogenesis of metabolic syndrome, including abdominal obesity, insulin resistance, dyslipidemia, and hypertension [15,16,17]. The role of HU as a cardiovascular risk factor has been firmly supported by a growing number of observational and mechanistic studies. A meta-analysis including over 340,000 participants demonstrated that each 1 mg/dL increase in serum uric acid was associated with a significantly higher risk of coronary heart disease and all-cause mortality, with a particularly strong effect in women [18]. Another meta-analysis of nearly one million individuals confirmed HU as an independent risk factor for coronary morbidity and mortality [19]. Collectively, this body of evidence underscores that HU is not merely a consequence of metabolic dysfunction but may actively contribute to the initiation and progression of cardiovascular disease.

HU is increasingly recognized as a metabolic state that intersects with lipid metabolism, thereby amplifying cardiovascular risk. Dyslipidemia is frequently observed in individuals with high serum uric acid, and these disturbances are mediated by shared mechanisms such as visceral adiposity, insulin resistance, and uric acid-induced oxidative stress that impair hepatic and vascular lipid handling [20,21,22]. To capture this complex interplay more effectively, recent studies have emphasized composite indices that integrate lipid fractions with markers of insulin resistance. The triglyceride–glucose (TyG) index, a validated surrogate for insulin resistance, has been shown to predict cardiovascular events, with individuals in the highest tertile demonstrating over twofold higher risk compared to those in the lowest [23]. Remnant cholesterol (RC), representing triglyceride-rich lipoproteins, has been causally linked to atherosclerosis and ischemic heart disease [24], while non-HDL-C provides a broader measure of cumulative atherogenic burden and is considered superior to LDL cholesterol for cardiovascular risk prediction [25]. In addition, indices such as the atherogenic index of plasma (AIP) and Castelli’s risk indices I and II (CRI-I and CRI-II) are established predictors of atherosclerotic cardiovascular disease [26]. Building on this global evidence, it becomes essential to contextualize the relationship between uric acid and lipid-related indices within specific populations, particularly in regions undergoing rapid metabolic and cardiovascular transitions.

Despite extensive global research linking HU to dyslipidemia and insulin resistance, important gaps remain regarding how uric acid relates to newer lipid-derived cardiometabolic indices, particularly TyG index, non-HDL-C, RC, AIP, and CRI-I and CRI-II. These indices capture metabolic risk more comprehensively than traditional lipids, yet very few studies have investigated their relationship with HU, and almost none have done so in Middle Eastern or Saudi populations. The Saudi population has markedly high rates of obesity, metabolic syndrome, and insulin resistance, suggesting a potentially stronger or distinct pattern of association between HU and these indices. However, existing local studies have focused primarily on conventional lipid markers and have not assessed whether uric acid correlates with lipid-derived cardiometabolic markers. The absence of regional data limits both the clinical utility of uric acid as a cardiometabolic marker in Saudi adults and our understanding of the nature of its association with lipid-derived cardiometabolic indices, thereby restricting the development of population-specific risk-stratification strategies.

This study aimed to provide a comprehensive evaluation of the relationship between HU and multiple lipid-derived cardiometabolic indices, mainly the TyG index, non-HDL-C, RC, AIP, CRI-I and CRI-II. To achieve this, we examined multivariable associations, assessed the discriminatory performance of serum uric acid for identifying individuals with elevated cardiometabolic indices using receiver operating characteristic (ROC) analysis, and quantified both the prevalence and odd ratios for adverse lipid-related phenotypes among HU adults.

## 2. Methods

### 2.1. Data Collection and Study Design

**Figure 1:**
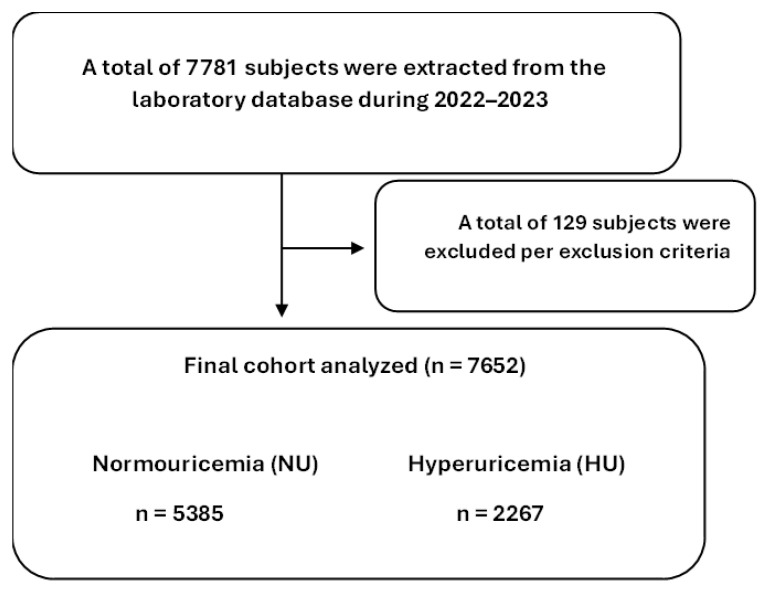
Flow diagram of study population selection.

This retrospective cross-sectional, single-center study analyzed data extracted from the Elta Medical Laboratory database between May and July 2025. The dataset consisted of routine laboratory samples originally collected from adult patients (≥18 years) who attended periodic checkups or were referred by physicians during 2022–2023. The Institutional Review Board of King Saud University, Riyadh, Saudi Arabia, approved the study protocol and design (Approval No: E-25-9893). Because the analysis was conducted on de-identified routine laboratory data, the need for informed consent was exempted. All assays were performed in a CAP-accredited laboratory environment following standardized quality control protocols. The study was conducted and reported in accordance with the STROBE (Strengthening the Reporting of Observational Studies in Epidemiology) guidelines. The completed STROBE checklist is available in the Supplementary File (Table S1). Inclusion criteria required complete laboratory profiles containing serum uric acid, lipid parameters, and relevant biochemical tests. Exclusion criteria included abnormal kidney function (elevated creatinine or urea based on laboratory reference intervals), missing data, and age below 18 years. After applying these criteria, 7652 participants remained from an initial 7781 identified records (Figure 1). Subjects were classified into normouricemic (NU) and hyperuricemic (HU) groups using sex-specific thresholds established for the Saudi population, with cut-offs of >7.46 mg/dL (444 µmol/L) for males and >5.40 mg/dL (321 µmol/L) for females, based on population-specific reference intervals validated in Saudi cohorts [27]. Atherogenic Index of Plasma (AIP) was calculated as log_10_(TG/HDL-C), with values >0.24 indicating high atherogenic risk [28]. Cardiovascular Risk Index I (CRI-I), defined as TC/HDL-C, and CRI-II, defined as LDL-C/HDL-C, were classified as elevated using established cut-offs of >5 and >3, respectively [28]. Non-HDL cholesterol was considered elevated at ≥150 mg/dL [29], while RC was classified as high at ≥24 mg/dL [30]. The TyG index, used as a surrogate marker of insulin resistance, was considered elevated at values > 4.72 [31]. These established reference cut-offs were applied uniformly across all analyses; ROC-derived thresholds are presented only as model outputs that illustrate the discriminatory capacity of uric acid and were not used to define elevated indices.

For each subject, demographic information, hematological and biochemical parameters, markers were retrieved, including red and white blood cell counts, fasting plasma glucose, renal profile and full lipid profile. The formulas used to derive cardiometabolic indices are outlined below. Detailed working examples of each equation are provided in Supplementary Table S2 for transparency and reproducibility.

(1) TyG=ln [TG (mg/dL)×FBG (mg/dL) / 2]

(2) Non−HDL=Total Cholesterol−HDL−C

(3) RC=Total Cholesterol−(LDL−C+HDL−C)

(4) $$AIP=Log10(\frac{TG}{HDL-C})$$

(5) $$CRI-I=\frac{Total\;Cholesterol}{HDL-C}$$

(6) $$CRI-II=\frac{LDL-C}{HDL-C}\;$$

### 2.2. Statistical Analysis

All analyses were performed using GraphPad Prism version 9.2.0 (GraphPad Software, Inc., San Diego, CA, USA). The distribution of continuous variables was assessed for normality with Kolmogorov–Smirnov and D’Agostino–Pearson tests. Given the comparison between two independent groups, Mann–Whitney U tests were used for continuous variables, with results presented as medians and interquartile ranges. Effect sizes (r) were calculated to quantify the magnitude of differences. Associations between HU and lipid-related cardiometabolic indices (TyG, non-HDL-C, RC, AIP, CRI-I, and CRI-II) were assessed using multivariable linear regression applied to the log-transformed variables. Four models were evaluated: unadjusted (Model 1), age- and sex-adjusted (Model 2), renal-adjusted (creatinine and urea; Model 3), and liver-adjusted (ALT and AST; Model 4). Regression coefficients (β) with 95% confidence intervals (CIs) were reported. Multicollinearity was examined using variance inflation factors (VIFs), which indicated no concerning collinearity. Receiver operating characteristic (ROC) curves were constructed to evaluate the discriminatory ability of serum uric acid for elevated cardiometabolic indices, with the area under the curve (AUC) and corresponding 95% CIs reported. Differences in the prevalence of elevated cardiometabolic indices between NU and HU groups were assessed using the Chi-square test of independence. Counts were derived from group-specific prevalence percentages and total sample sizes. Prevalence ratios (RRs) and odds ratios (ORs) with 95% CIs were calculated using 2 × 2 contingency tables in MedCalc (MedCalc Software Ltd., Ostend, Belgium; https://www.medcalc.org/en/calc/relative_risk.php, accessed on 25 May 2025). Binary variables (elevated vs. non-elevated indices) were defined using established cut-offs. All reported *p*-values were corrected for multiple comparisons using the Benjamini–Hochberg false discovery rate (FDR) procedure. Statistical significance was defined as *p* < 0.05.

## 3. Results

### 3.1. Baseline Biochemical and Hematological Characteristics by Uricemia Status

Compared with the NU group (Table 1), individuals with HU exhibited a more atherogenic and metabolically adverse profile. HU subjects had significantly higher triglycerides (107 vs. 88 mg/dL, *p* = 0.004) and lower HDL cholesterol (44 vs. 49 mg/dL, *p* < 0.001), indicating unfavorable lipid alterations. Markers of hepatic function were also higher among HU individuals, including ALT (23 vs. 16 U/L, *p* < 0.001) and AST (20 vs. 17 U/L, *p* = 0.014). Additionally, HU individuals demonstrated higher urea levels (22 vs. 21 mg/dL, *p* = 0.014). Other parameters including age, red cell count, total leukocyte count, LDL-C, total cholesterol, fasting glucose, and creatinine did not differ significantly between groups (*p* > 0.999).

### 3.2. Sex and Age-Specific Analysis in Cardiometabolic Indices by Uricemia Status

**Figure 2:**
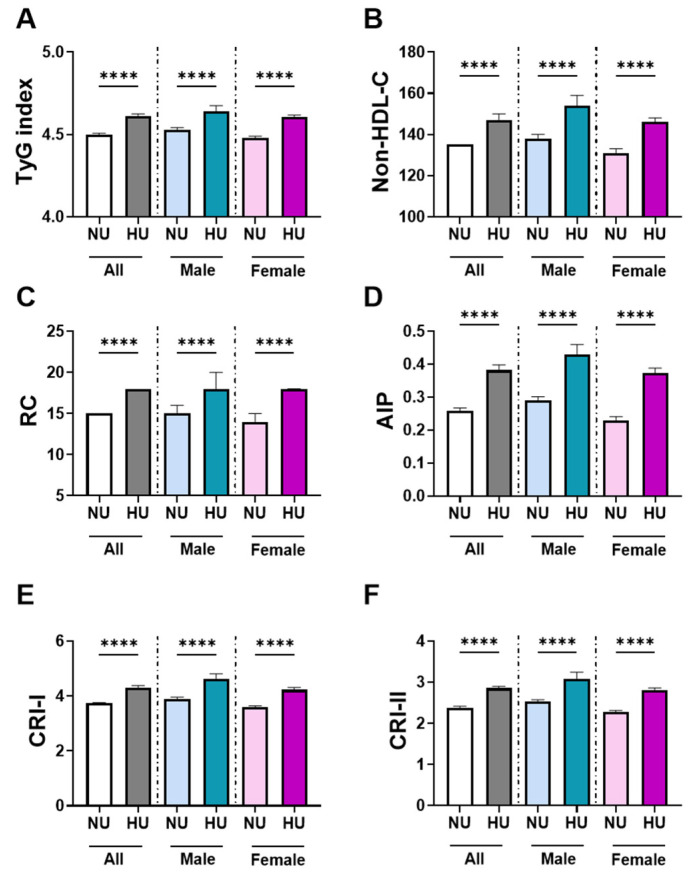
Comparison of cardiometabolic indices between normouricemia (NU) and hyperuricemia (HU). Each row of bar graphs displays results for the total population (left), male subgroup (middle), and female subgroup (right). Panels represent: (**A**) triglyceride–glucose index (TyG), (**B**) non-high-density lipoprotein cholesterol (Non-HDL-C), (**C**) remnant cholesterol (RC), (**D**) atherogenic index of plasma (AIP), (**E**) Castelli risk index I (CRI-I), and (**F**) Castelli risk index II (CRI-II). Data are shown as median and interquartile range (IQR). **** indicates *p* < 0.0001.

Stratified analyses revealed consistent sex- and age-specific patterns in the distribution of cardiometabolic indices across NU and HU groups. TyG values (Figure 2A) were higher in the HU group (4.610, 4.446–4.771) than in the NU group (4.500, 4.349–4.668), with a small effect size (Cliff’s δ = −0.241). Non-HDL-C (Figure 2B) was similarly elevated among HU subjects (147, 120–171 mg/dL) compared with NU individuals (135, 115–156 mg/dL) (δ = −0.179). RC (Figure 2C) also showed higher levels in HU (18, 12–26 mg/dL) than NU (15, 10–21.5 mg/dL) (δ = −0.191). Atherogenic indices showed the strongest differences: AIP (Figure 2D; 0.3819, 0.2258–0.5518 vs. 0.2580, 0.0969–0.4301; δ = −0.281), CRI-I (Figure 2E; 4.300, 3.568–5.128 vs. 3.736, 3.138–4.465; δ = −0.280), and CRI-II (Figure 2F; 2.854, 2.224–3.545 vs. 2.383, 1.870–3.018; δ = −0.260).

Sex-stratified analyses showed consistent patterns with varying magnitudes. Among males, HU participants had higher TyG (4.641, 4.474–4.805 vs. 4.527, 4.370–4.697; δ = −0.24), Non-HDL-C (154, 114–183 mg/dL vs. 138, 116–160 mg/dL; δ = −0.19), RC (18, 13–26 mg/dL vs. 15, 10–22 mg/dL; δ = −0.18), AIP (0.4286, 0.2576–0.5976 vs. 0.2903, 0.1298–0.4664; δ = −0.29), CRI-I (4.627, 3.723–5.481 vs. 3.892, 3.250–4.683; δ = −0.29), and CRI-II (3.075, 2.344–3.836 vs. 2.525, 1.969–3.190; δ = −0.28). In females, differences were slightly more pronounced. HU women had higher TyG (4.605, 4.442–4.765 vs. 4.480, 4.328–4.638; δ = −0.29), Non-HDL-C (146, 120.8–169 mg/dL vs. 131, 113–153 mg/dL; δ = −0.21), RC (18, 12–26 mg/dL vs. 14, 9–21 mg/dL; δ = −0.22), AIP (0.3739, 0.2144–0.5422 vs. 0.2282, 0.068–0.387; δ = −0.33), CRI-I (4.230, 3.543–5.025 vs. 3.603, 3.043–4.243; δ = −0.34), and CRI-II (2.810, 2.199–3.456 vs. 2.273, 1.794–2.829; δ = −0.32).

**Figure 3:**
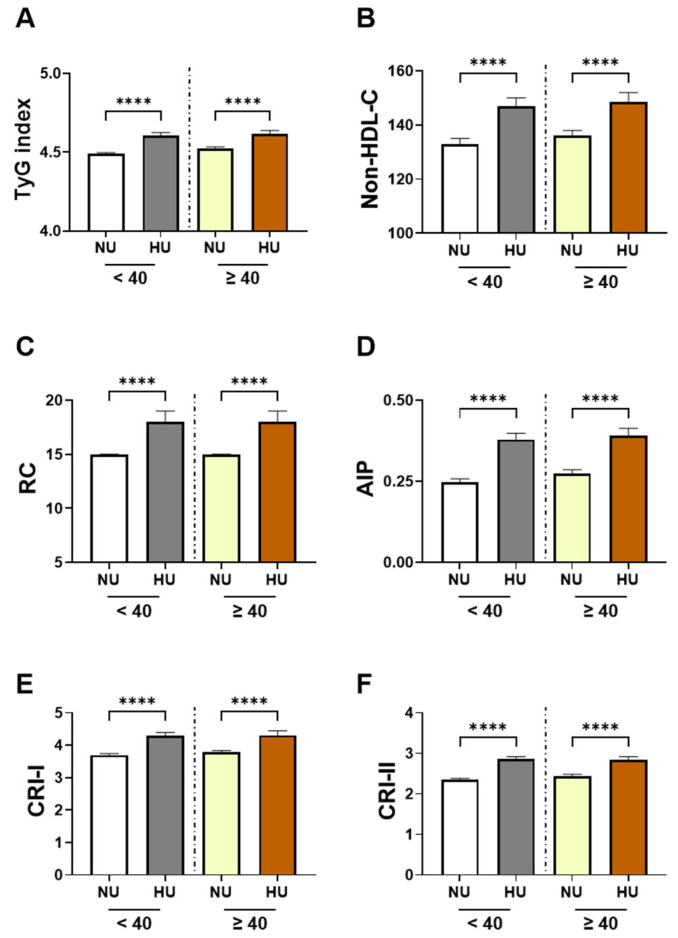
Distribution of cardiometabolic indices stratified by age group and uricemia status. Each row of bar graphs displays results for younger subjects (<40 years; left) and older subjects (≥40 years; right), stratified into normouricemic (NU) and hyperuricemic (HU) subgroups. Panels represent: (**A**) triglyceride–glucose index (TyG), (**B**) non-high-density lipoprotein cholesterol (Non-HDL-C), (**C**) remnant cholesterol (RC), (**D**) atherogenic index of plasma (AIP), (**E**) Castelli risk index I (CRI-I), and (**F**) Castelli risk index II (CRI-II). Data are shown as median and interquartile range (IQR). Ns indicates not significant while **** indicates *p* < 0.0001.

When stratified by age (<40 years vs. ≥40 years), individuals with HU consistently exhibited higher levels of all cardiometabolic indices compared with their NU counterparts (Figure 3). Among participants younger than 40 years, HU individuals showed higher TyG values (4.607, 4.441–4.764 vs. 4.489, 4.339–4.657; δ = −0.253). Similar differences were observed for Non-HDL-C (147, 119–170.5 mg/dL vs. 133, 114–155 mg/dL; δ = −0.184), RC (18, 12–26 mg/dL vs. 15, 10–21 mg/dL; δ = −0.201), and AIP (0.3791, 0.2273–0.5506 vs. 0.2478, 0.0882–0.4151; δ = −0.295). CRI-I (4.295, 3.557–5.162 vs. 3.696, 3.109–4.438; δ = −0.295) and CRI-II (2.854, 2.217–3.540 vs. 2.355, 1.843–3.000; δ = −0.272) also demonstrated moderately sized negative Cliff’s delta values.

In participants aged 40 years and older, the same pattern persisted with slightly smaller effect sizes. HU adults had higher TyG (4.615, 4.454–4.783 vs. 4.522, 4.364–4.685; δ = −0.228), Non-HDL-C (148.5, 121.8–172.0 mg/dL vs. 136, 116–158 mg/dL; δ = −0.175), and RC (18, 12–26 mg/dL vs. 15, 10–22 mg/dL; δ = −0.176). Differences in AIP (0.3906, 0.2254–0.5536 vs. 0.2730, 0.1161–0.4449; δ = −0.263), CRI-I (4.305, 3.582–5.073 vs. 3.783, 3.203–4.500; δ = −0.259), and CRI-II (2.846, 2.238–3.565 vs. 2.442, 1.925–3.048; δ = −0.243).

### 3.3. Multivariable Associations Between HU and Cardiometabolic Indices

In Table 2, regression analyses demonstrated that HU was significantly associated with all cardiometabolic indices in the unadjusted model (Model 1). Across all four independently fitted regression models, HU showed positive associations with all cardiometabolic indices examined. In unadjusted analyses, the strongest associations were observed for CRI-I (β = 0.37, 95% CI: 0.34–0.40), CRI-II (β = 0.24, 95% CI: 0.22–0.26), and AIP (β = 0.18, 95% CI: 0.17–0.20). These associations remained statistically significant in all adjusted models, whether controlling for age and sex (Model 2), renal markers (Model 3), or liver enzymes (Model 4). The only exception was non-HDL-C, for which the association became non-significant in the liver-adjusted model (β = 0.01, 95% CI: −0.02 to 0.05). TyG demonstrated the greatest attenuation across models but remained significant in all except the liver profile-adjusted model. Variance inflation factors (VIF) were consistently near 1, indicating no evidence of multicollinearity.

The largest effect sizes were observed for CRI-I (β = 0.37, 95% CI: 0.34–0.40, *p* < 0.0001) and CRI-II (β = 0.24, 95% CI: 0.22–0.26, *p* < 0.0001), followed by AIP (β = 0.18, 95% CI: 0.17–0.20, *p* < 0.0001) and TyG (β = 1.58, 95% CI: 1.41–1.76, *p* < 0.0001). Adjustment for age and sex (Model 2) resulted in minimal change in the coefficients, indicating limited confounding by demographic factors. After further adjustment for kidney profile (Model 3), effect sizes decreased across most indices; TyG showed a notable reduction (β = 1.17, 95% CI: 0.97–1.37, *p* < 0.0001), while associations for CRI-I (β = 0.26, 95% CI: 0.22–0.30, *p* < 0.0001), CRI-II (β = 0.17, 95% CI: 0.14–0.20, p < 0.0001), and AIP (β = 0.13, 95% CI: 0.11–0.15, *p* < 0.0001) remained statistically significant. In the model adjusted for liver profile parameters (Model 4), significant associations persisted for RC (β = 0.05, 95% CI: 0.04–0.06, *p* < 0.0001), AIP (β = 0.13, 95% CI: 0.11–0.14, *p* < 0.0001), CRI-I (β = 0.23, 95% CI: 0.20–0.26, *p* < 0.0001), and CRI-II (β = 0.14, 95% CI: 0.11–0.16, *p* < 0.0001). By contrast, the association with Non-HDL-C lost significance (β = 0.01, 95% CI: −0.02–0.05, *p* = 0.48), and effect size of TyG was attenuated (β = 0.94, 95% CI: 0.77–1.12, *p* < 0.0001).

### 3.4. Discrimnatory Performance of HU for Cardiometabolic Indices

**Figure 4:**
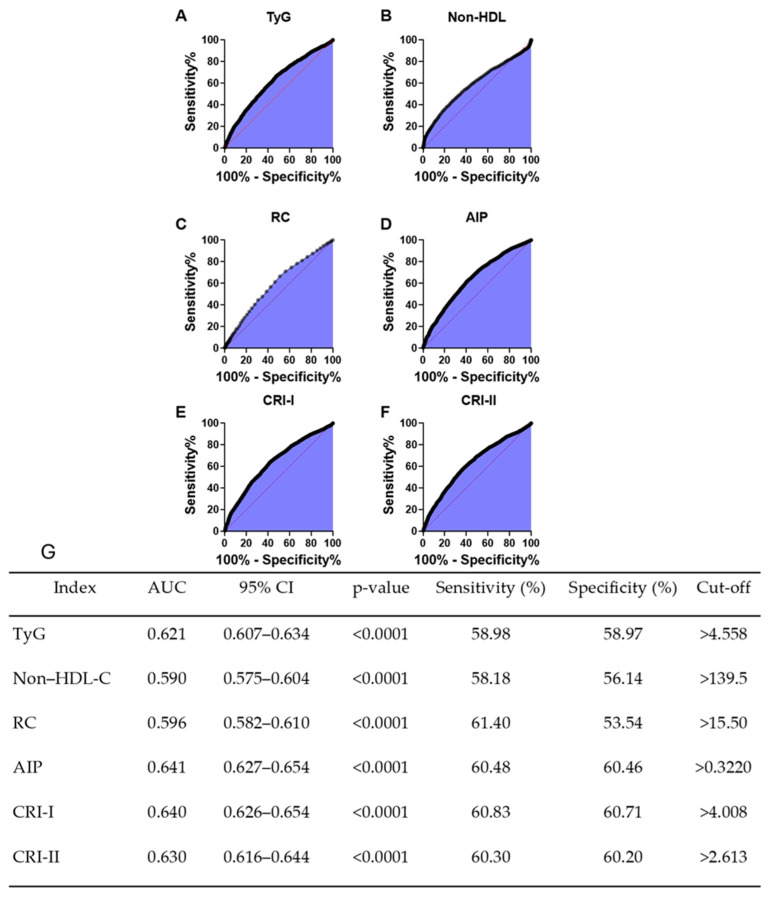
Receiver operating characteristic (ROC) curves showing the ability of serum uric acid to predict elevated cardiometabolic indices. The red diagonal line on each plot represents the reference null classifier (AUC 0.5). Plots show the diagnostic performance of (**A**) triglyceride–Glucose index (TyG), (**B**) non-high density lipoprotein cholesterol (non-HDL-C), (**C**) remnant cholesterol (RC), (**D**) Atherogenic Index of Plasma (AIP), (**E**) Castelli’s Risk Index I (CRI-I), and (**F**) Castelli’s Risk Index II (CRI-II). Panel (**G**) provides the corresponding summary table of AUC values, 95% CIs, optimal cut-off points, sensitivity, and specificity.

ROC curve analysis demonstrated that serum uric acid significantly discriminated against individuals with elevated cardiometabolic indices (Figure 4). The discriminatory capacity, however, was of modest magnitude across all indices. Among the markers evaluated, the strongest predictive performance was observed for the atherogenic indices AIP (AUC = 0.641) and CRI-I (AUC = 0.640), followed closely by CRI-II (AUC = 0.630). In comparison, the ability of uric acid to discriminate elevated TyG (AUC = 0.621) and RC (AUC = 0.596) was modest, while the weakest predictive value was observed for Non-HDL-C(AUC = 0.590). Although statistically significant, these AUC values fall within the range considered modest discriminatory performance (<0.70).

### 3.5. Prevalence of Elevated Cardiometabolic Indices in HU

The prevalence of elevated cardiometabolic indices was consistently higher among HU individuals compared with NU subjects (Table 3). Elevated TyG was observed in 32.9% of HU versus 18.3% of NU individuals, while elevated Non-HDL-C was present in 48.2% versus 32.3%, respectively. Similarly, elevated RC (31.1% vs. 20.5%) and AIP (73.3% vs. 53%) were more frequent in the HU group. Notably, composite risk ratios showed the greatest differences: CRI-I was elevated in 28.2% of HU compared with 13.7% of NU, and CRI-II in 44.0% versus 25.9%, respectively.

### 3.6. HU-Associated Prevalence Estimates for Cardiometabolic Indices

**Figure 5:**
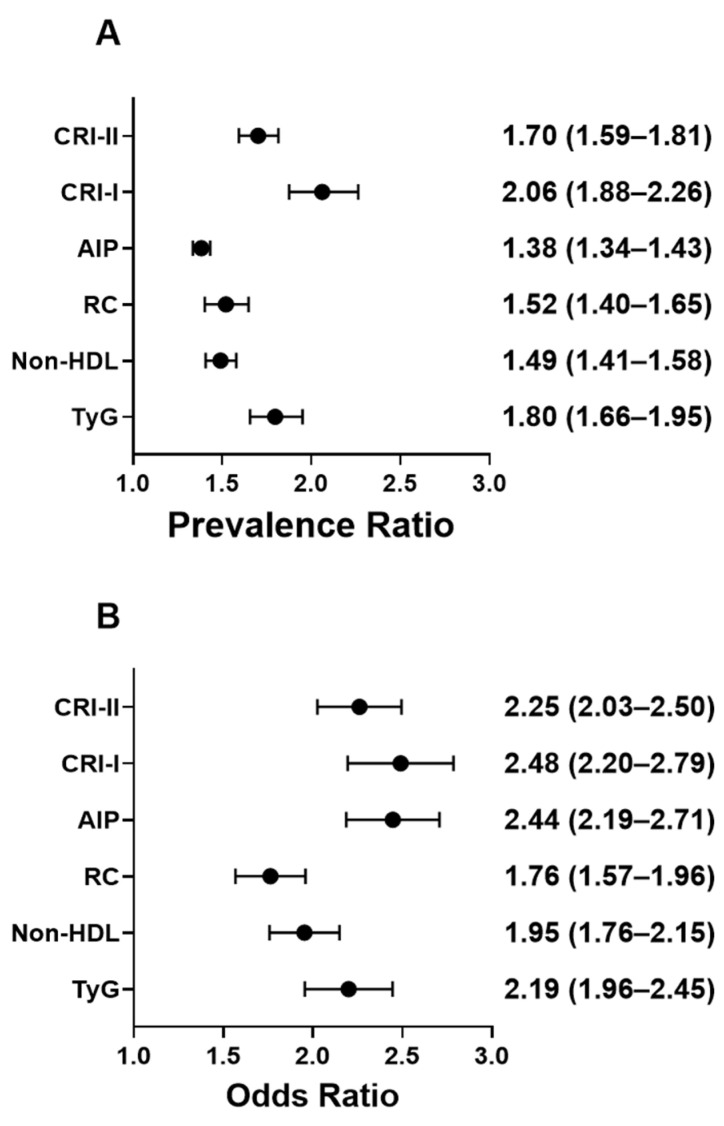
Prevalence ratios and Odds ratios for lipid-derived cardiometabolic indices in relation to hyperuricemia. Forest plots present (**A**) prevalence ratio and (**B**) odds ratio with corresponding 95% confidence intervals (CIs) for triglyceride–Glucose index (TyG), non-high density lipoprotein cholesterol (non-HDL), remnant cholesterol (RC), Atherogenic Index of Plasma (AIP), and Castelli’s Risk Indices I (CRI-I) and II (CRI-II).

Association analyses indicated that HU was consistently linked with a higher prevalence of elevated cardiometabolic indices (Figure 5). Prevalence ratios showed the strongest associations for CRI-I (PR: 2.06, 95% CI: 1.88–2.26, *p* < 0.0001) and TyG (PR: 1.80, 95% CI: 1.66–1.95, *p* < 0.0001), followed by CRI-II (PR: 1.70, 95% CI: 1.59–1.81, *p* < 0.0001), RC (PR: 1.52, 95% CI: 1.40–1.65, *p* < 0.0001), non- HDL-C (PR: 1.49, 95% CI: 1.41–1.58, *p* < 0.0001), and AIP (PR: 1.38, 95% CI: 1.34–1.43, *p* < 0.0001). Odds ratios demonstrated similar patterns, with markedly higher odds of elevated indices among HU individuals, particularly for CRI-I (OR: 2.48, 95% CI: 2.20–2.79, *p* < 0.0001), AIP (OR: 2.44, 95% CI: 2.19–2.71, *p* < 0.0001) and CRI-II (OR: 2.25, 95% CI: 2.03–2.50, *p* < 0.0001). Additional associations were observed for TyG (OR: 2.19, 95% CI: 1.96–2.45, *p* < 0.0001), non-HDL-C (OR: 1.95, 95% CI: 1.76–2.15, *p* < 0.0001), and RC (OR: 1.76, 95% CI: 1.57–1.96, *p* < 0.0001).

## 4. Discussion

In this large cohort, HU was consistently associated with a range of adverse cardiometabolic abnormalities. Individuals with elevated uric acid levels had higher TG along with lower HDL-C. Importantly, HU showed a statistically significant but modest association with the TyG index, a surrogate of insulin resistance, as well as to RC, non-HDL-C, AIP, CRI-I and CRI-II. Prevalence ratio and odds ratio analyses reinforced these associations, showing that individuals with HU had a substantially higher prevalence, and markedly greater odds, of exhibiting elevated values across all cardiometabolic indices. The strongest relationships were observed for CRI-I, CRI-II, and AIP, which consistently demonstrated the highest prevalence and odds ratios and are well-established markers of atherogenic dyslipidemia and cardiovascular risk. ROC curve analyses further demonstrated that HU was associated with cardiometabolic indices that exhibited modest yet significant discriminatory power in distinguishing individuals with elevated risk. Collectively, these results highlight that HU extends far beyond purine metabolism or gout-related pathology and is linked with atherogenic lipid abnormalities, insulin resistance, and markers of vascular dysfunction. These findings provide the first evidence of these associations in a large Saudi cohort, documenting previously unreported links between uric acid levels and multiple cardiometabolic indices. These findings remain descriptive rather than causal, and further longitudinal studies are needed to clarify their temporal and biological context.

HU individuals demonstrated significantly higher TG accompanied by lower HDL-C compared with NU adults. These findings are particularly relevant since they contribute directly to composite lipid indices such as AIP, CRI-I, and CRI-II indices that were the primary focus of this work. Thus, the baseline contrasts support the study aim by showing that HU is associated with multiple lipid components that directly underpin the composite cardiometabolic indices evaluated. Furthermore, stratified analyses demonstrated that the associations between HU and elevated cardiometabolic indices were consistent across sex and age groups. In both males and females, and in both younger (<40 years) and older (≥40 years) adults, HU was associated with higher TyG, non-HDL-C, RC, AIP, CRI-I, and CRI-II. Evidence from multiple populations supports the notion that sex does not largely alter the direction of the relationship between uric acid and lipid abnormalities. In young adults from Bangladesh, serum uric acid showed significant positive associations with TG, LDL-C, and overall dyslipidemia in both males and females [32]. Likewise, in high-altitude Naxi adults, the TG/HDL-C ratio was positively associated with HU in both men and women [33]. These findings show that despite sex differences in absolute serum uric acid levels and HU prevalence, the association between hyperuricemia and lipid-derived cardiometabolic indices remains directionally consistent across sexes, suggesting that uric acid might interact with lipid metabolism similarly in men and women.

Mechanistic studies provide important insights into the relationship between HU and adverse cardiovascular outcomes. Xanthine oxidoreductase (XOR), particularly in its oxidase form, accumulates in atherosclerotic plaques where it generates reactive oxygen species (ROS), promoting endothelial injury, vascular smooth muscle cell proliferation, and upregulation of monocyte chemotactic protein-1 (MCP-1), thereby driving inflammation and plaque progression [34,35]. Furthermore, experimental work has shown that XOR activity contributes to macrophage foam cell formation, while XOR knockdown reduces lipid uptake and inflammatory cytokine production, linking uric acid metabolism directly to dyslipidemia [36]. Clinical studies reinforce these findings; in symptomatic patients with carotid atherosclerosis, XO expression was markedly elevated and correlated positively with serum uric acid while inversely associated with HDL-C, further supporting the role of uric acid metabolism in vascular lipid abnormalities [37]. Collectively, this body of evidence suggests that elevated uric acid and XOR activity promote oxidative stress, lipid accumulation, and systemic inflammation, offering a plausible explanation for the strong associations observed in our study between HU and atherogenic lipid indices, insulin resistance, and other cardiometabolic risk markers.

Several lines of evidence support the association between HU and insulin resistance, as reflected by the elevated TyG index in our study. Experimental data demonstrate that soluble uric acid enhances NALP3 inflammasome activation and IL-1β expression in human renal proximal tubular epithelial cells via a TLR4-dependent pathway [38]. In parallel, renal NF-κB activation has been shown to disrupt uric acid homeostasis and drive tumor-associated mortality independently of wasting [39]. Moreover, uric acid has been reported to stimulate NF-κB activation and inflammatory signaling in the hypothalamus [40], thereby providing a mechanistic link between uric acid, systemic metabolic disturbances. Clinical studies further corroborate these mechanistic insights, showing that higher uric acid levels are associated with increased circulating IL-6 and IL-1β in hospitalized older adults [41] and with elevated IL-6 and TNF-α but lower IL-1β in community cohorts [42]. Mechanistic research also demonstrates that HU modulates macrophage polarization toward a pro-inflammatory M1 phenotype, enhancing cytokine release and impairing insulin sensitivity, while suppressing the protective, anti-inflammatory M2 response [43,44]. Clinically, uric acid-lowering therapy with allopurinol has been shown to improve insulin resistance, reduce inflammatory markers, and attenuate atherosclerosis progression in patients with type 2 diabetes and asymptomatic HU [45,46]. Collectively, these findings provide a plausible explanation for our observation that HU is closely associated with elevated TyG index, supporting the notion that uric acid contributes both to insulin resistance and to cardiometabolic dysfunction through intertwined inflammatory and metabolic pathways.

RC and non-HDL-C have been reported in prior studies to correlate with HU and various metabolic disturbances. Previous literature suggests that these lipid indices frequently coexist with alterations in lipid metabolism, purine metabolism, insulin resistance, and renal function [47]. RC plays a central role in lipid transport and, when elevated, induces increased turnover of free fatty acids (FFAs) [47]. This heightened flux of FFAs accelerates the breakdown of adenosine triphosphate (ATP), leading to greater purine degradation and subsequently higher serum uric acid levels [48,49]. Beyond this metabolic pathway, RC has been shown to independently associate with a reduced estimated glomerular filtration rate (eGFR) and a heightened risk of renal impairment [50,51]. Because the kidneys are responsible for the majority of uric acid excretion, impaired renal function further aggravates uric acid retention. Importantly, RC is also regarded as a surrogate marker of insulin resistance (IR), a well-established factor in the pathogenesis of HU [52]. IR contributes to decreased renal clearance of uric acid, while simultaneously promoting lipotoxicity and systemic inflammation, thereby creating a vicious cycle between dyslipidemia and HU [48]. Non-HDL-C, which encompasses RC and other atherogenic lipoproteins such as VLDL, IDL, and LDL, provides a broader measure of atherogenic burden and captures the cumulative effect of lipid abnormalities on uric acid metabolism [25]. Its elevation reflects overlapping mechanisms of lipid overload, oxidative stress, renal dysfunction, and IR that converge to elevate uric acid concentrations [53,54]. In the context of the present study, individuals with HU demonstrated higher levels of RC and non-HDL-C. While these findings are consistent with previously described associations in other populations, they do not establish mechanistic relationships or clinical implications. Instead, they provide descriptive evidence of co-occurring abnormalities in lipid indices among individuals with elevated uric acid levels. Given the cross-sectional design and the modest effect sizes observed, no conclusions can be drawn regarding clinical utility or underlying biological pathways. The potential relevance of RC and non-HDL-C in HU states should be examined in future longitudinal studies.

The present findings indicate that serum uric acid is associated with several adverse lipid-derived cardiometabolic indices in this cohort. Although these associations were modest, they suggest that higher uric acid levels may coexist with broader patterns of metabolic dysregulation. Rather than implying a clinical or predictive role, these results provide descriptive evidence that uric acid correlates with markers commonly involved in cardiometabolic risk. Individuals with HU often present with comorbid conditions such as hypertension, metabolic syndrome, and chronic kidney disease, as shown in previous literature [55,56]. The current findings add to this body of evidence by documenting co-occurrence patterns, but they do not support using uric acid for risk prediction or stratification. Further longitudinal studies with comprehensive clinical and lifestyle data are required to clarify temporal relationships and determine whether uric acid contributes to cardiometabolic risk assessment.

Several constraints of this work should be considered when interpreting the findings. Because of its retrospective and cross-sectional nature, the study cannot determine causal relationships or clarify whether HU precedes or results from the observed cardiometabolic disturbances. Because the dataset originated from a single-center laboratory, the findings may not fully reflect broader population variability. Although adjustments were made for key demographic and biochemical covariates, important clinical confounders including BMI, blood pressure, glycemic status/diabetes, and other cardiometabolic determinants were not available in the dataset and therefore could not be incorporated into the analytical models, raising the possibility of unmeasured or residual confounding. In addition, lifestyle-related factors such as dietary patterns, physical activity, alcohol consumption, comorbidities and the use of medications that affect uric acid or lipid metabolism, were also unavailable. Finally, as the cohort consisted exclusively of Saudi adults, the findings may not be directly generalizable to populations with different ethnic or environmental backgrounds.

## 5. Conclusions

In this large cross-sectional cohort, HU was modestly yet consistently associated with several lipid-derived cardiometabolic indices, including atherogenic ratios and insulin-resistance-related markers. These associations, observed across sex and age groups, suggest that elevated uric acid levels coexist with an adverse cardiometabolic profile, although the strength of the relationships was generally limited. Given the increasing prevalence of HU and metabolic disorders in Saudi adults, uric acid may represent a complementary marker within broader cardiometabolic assessments. However, longitudinal studies are needed to elucidate temporal relationships and to determine whether modifying uric acid levels influences lipid parameters or cardiometabolic outcomes.

## Figures and Tables

**Table 1:** Baseline Biochemical and Hematological Characteristics of study subjects by Uricemia Status.

Variable	Normouricemia	Hyperuricemia	*p* Value
Age (years)	38 (32–46)	36 (32–45)	>0.999
Red cell count (×10^6^/µL)	5.14 (4.80–5.54)	5.53 (5.09–5.89)	0.1318
Total leucocytic count (×10^3^/µL)	5.57 (4.49–6.90)	5.86 (4.73–7.15)	>0.999
Triglycerides (mg/dL)	88 (67–120)	107 (79–144)	0.0040
HDL cholesterol (mg/dL)	49 (42–57)	44 (37–51)	<0.001
LDL cholesterol (mg/dL)	117 (99–137)	127 (101–149)	>0.999
Total cholesterol (mg/dL)	185 (165–206)	193 (165–216)	>0.999
Fasting plasma glucose (mg/dL)	91 (85–98)	94 (88–102)	>0.999
Creatinine (mg/dL)	0.70 (0.60–0.80)	0.80 (0.70–0.98)	0.4543
Urea (mg/dL)	21 (17–27)	22 (11–29)	0.0142
Alanine Aminotransferase (U/L)	16 (12–24)	23 (16–33)	<0.001
Aspartate Aminotransferase (U/L)	17 (15–22)	20 (16–25)	0.0142

**Table 2:** Multivariable Associations Between HU and Cardiometabolic Indices.

Model	Index	β (95% CI)	VIF	R^2^	*p* Value
Model 1	TyG	1.58 (1.41–1.76)	1.00	0.040	<0.0001
	Non-HDL-C	0.14 (0.11–0.18)	1.00	0.007	<0.0001
	RC	0.09 (0.07–0.10)	1.00	0.022	<0.0001
	AIP	0.18 (0.17–0.20)	1.00	0.065	<0.0001
	CRI-I	0.37 (0.34–0.40)	1.00	0.059	<0.0001
	CRI-II	0.24 (0.22–0.26)	1.00	0.045	<0.0001
Model 2	TyG	1.57 (1.40–1.74)	1.00	0.042	<0.0001
	Non-HDL-C	0.14 (0.10–0.18)	1.00	0.012	<0.0001
	RC	0.09 (0.07–0.10)	1.00	0.025	<0.0001
	AIP	0.18 (0.17–0.20)	1.00	0.067	<0.0001
	CRI-I	0.36 (0.33–0.40)	1.00	0.062	<0.0001
	CRI-II	0.24 (0.21–0.26)	1.00	0.048	<0.0001
Model 3	TyG	1.17 (0.97–1.37)	1.03	0.317	<0.0001
	Non-HDL-C	0.09 (0.05–0.13)	1.04	0.302	<0.0001
	RC	0.06 (0.04–0.07)	1.03	0.306	<0.0001
	AIP	0.13 (0.11–0.15)	1.06	0.324	<0.0001
	CRI-I	0.26 (0.22–0.30)	1.10	0.321	<0.0001
	CRI-II	0.17 (0.14–0.20)	1.08	0.316	<0.0001
Model 4	TyG	0.94 (0.77–1.12)	1.11	0.105	<0.0001
	Non-HDL-C	0.01 (–0.02–0.05)	1.08	0.092	0.48
	RC	0.05 (0.04–0.06)	1.05	0.100	<0.0001
	AIP	0.13 (0.11–0.14)	1.14	0.119	<0.0001
	CRI-I	0.23 (0.20–0.26)	1.17	0.112	<0.0001
	CRI-II	0.14 (0.11–0.16)	1.15	0.106	<0.0001

Model 1 represents unadjusted analysis with no covariates included. Model 2 adjusts only for age and sex. Model 3 adjusts exclusively for renal profiles, namely creatinine and urea. Model 4 adjusts solely for hepatic enzymes, namely AST and ALT. Abbreviations: TyG = triglyceride–glucose index; Non-HDL-C = non-high-density lipoprotein cholesterol; RC = remnant cholesterol; AIP = atherogenic index of plasma; CRI-I = Castelli’s risk index I; CRI-II = Castelli’s risk index II.

**Table 3:** Comparative Prevalence of Elevated Cardiometabolic Risk Indices Between Normouricemic (NU) and Hyperuricemic (HU) subjects.

Variable	NU (%)	HU (%)	*p* Value
Elevated TyG (>4.72)	18.3	32.9	<0.0001
Elevated Non-HDL-C (≥150 mg/dL)	32.3	48.2	<0.0001
Elevated RC (≥24 mg/dL)	20.5	31.1	<0.0001
Elevated AIP (>0.24)	53.0	73.3	<0.0001
Elevated CRI-I (>5)	13.7	28.2	<0.0001
Elevated CRI-II (>3)	25.9	44.0	<0.0001

Abbreviations: TyG, Triglyceride–Glucose index; Non-HDL-C, Non-high-density lipoprotein cholesterol; RC, Remnant cholesterol; AIP, Atherogenic Index of Plasma; CRI-I, Castelli’s Risk Index I; CRI-II, Castelli’s Risk Index II; NU, Normouricemia; HU, Hyperuricemia.

## Data Availability

The datasets generated and/or analyzed during the current study are not publicly available due to institutional restrictions and the requirement for authorization from Elta Medical Laboratories. Access to these data may be granted upon reasonable request to the corresponding author (Y.A.) and is subject to approval by Elta Medical Laboratories.

## Supplementary Materials

The following supporting information can be downloaded at: https://www.mdpi.com/article/10.3390/healthcare13233151/s1, Table S1: STROBE Checklist. Table S2: Worked Examples for Derived Cardiometabolic Indices.
