# Supplementary material for: Lipid-Derived Cardiometabolic Indices in Normouricemic and Hyperuricemic Adults: A Retrospective Cross-Sectional Association Study
Source: Healthcare (Basel). 2025 Dec 3;13(23):3151. doi: 10.3390/healthcare13233151 (PMC12691944; doi:10.3390/healthcare13233151)
Supplement: Supplementary file 1 [file healthcare-13-03151-s001.zip › healthcare-3941296-supplementary.pdf]

Supplementary Table S1. STROBE Checklist

Item	No.	Recommendation	Where Addressed in Manuscript
**Title and abstract**	**1**	(a) Indicate study design in title/abstract.<br>(b) Provide informative and balanced summary.	Title; Abstract
**Introduction**			
**Background/rationale**	**2**	Explain scientific background and rationale.	Introduction, paragraphs 1–2
**Objectives**	**3**	State specific objectives and hypotheses.	Introduction, final paragraph
**Methods**			
**Study design**	**4**	Present key elements of study design early.	Methods, first sentence ("retrospective cross-sectional study")
**Setting**	**5**	Describe setting, location, dates, recruitment, data collection.	Methods ("Elta Medical Laboratory"; samples 2022–2023; extraction May–July 2025)
**Participants**	**6**	(a) Eligibility criteria and methods of selection.	Methods ("inclusion/exclusion criteria")
**Variables**	**7**	Define outcomes, exposures, predictors, confounders; diagnostic criteria.	Methods (definitions of HU, lipid indices, TyG, atherogenic ratios)
**Data sources/measurement**	**8***	Describe data sources, measurement methods, comparability.	Methods (laboratory assays, equipment, QC; Saudi-specific uric acid cutoffs)
**Bias**	**9**	Efforts to address potential bias.	Methods (standardized QC; exclusion of incomplete data); Limitations
**Study size**	**10**	Explain how study size was arrived at.	Methods (initial 7,781 → final 7,652 after exclusions)
**Quantitative variables**	**11**	How quantitative variables handled; groupings chosen.	Methods (Statistical Analysis section).
**Statistical methods**	**12**	(a) Describe all statistical methods; confounding control.<br>(b) Subgroups/interactions.<br>(c) Missing data.<br>(d) Sampling strategy.<br>(e) Sensitivity analyses.	Methods (Statistical Analysis section).
**Results**			
**Participants**	**13***	(a) Report numbers at each stage.<br>(b) Reasons for non-participation.<br>(c) Use flow diagram (optional).	Methods and Figure 1.

**Descriptive data**	**14***	(a) Characteristics of participants; exposures, confounders.<br>(b) Missing data numbers.	Table 1; Methods (missing data handled via exclusion).
**Outcome data**	**15***	Report outcome events/summary measures.	Results; Tables 1–2
**Main results**	**16**	(a) Unadjusted & adjusted estimates with 95% CI; confounders listed.<br>(b) Category boundaries.<br>(c) Translate relative risk to absolute risk (if relevant).	Results; Table 2; Methods (cutoffs). RR not applicable (cross-sectional).
**Other analyses**	**17**	Report subgroup, interaction, and sensitivity analyses.	Figure 2,3,4 and Table 3
**Discussion**			
**Key results**	**18**	Summarize key results with reference to objectives.	Discussion, opening paragraph
**Limitations**	**19**	Discuss limitations, potential bias/imprecision; direction and magnitude.	Expanded Limitations section (single-center, narrow window, no diet/medications, residual confounding)
**Interpretation**	**20**	Provide cautious interpretation considering objectives, limitations, literature.	Discussion
**Generalisability**	**21**	Discuss external validity.	Discussion; Limitations
**Other information**			
**Funding**	**22**	Source and role of funders.	Funding Statement

Supplementary Table S2. Worked Examples for Derived Cardiometabolic Indices

The following example uses representative laboratory values to illustrate how each cardiometabolic index was calculated.
Values used: **TG = 150 mg/dL, HDL-C = 50 mg/dL, LDL-C = 120 mg/dL, Total Cholesterol = 190 mg/dL, FBG = 100 mg/dL**

Index	Formula	Substitution	Calculated Value
**TyG**	= ln [TG (mg/dL) × FBG (mg/dL) / 2]	ln[(150 × 100)/2] = ln(7,500)	8.92
**Non–HDL-C**	= TC − HDL-C	190 − 50	140 mg/dL
**RC**	= TC – (LDL-C + HDL-C)	190 – (120 – 50)	20 mg/dL
**AIP**	= $\log_{10}$(TG/HDL-C)	$\log_{10}$(150/50) = $\log_{10}$(3)	0.48
**CRI-I**	= TC / HDL-C	190 / 50	3.80
**CRI-II**	= LDL-C / HDL-C	120 / 50	2.40

Abbreviations: TyG = triglyceride-glucose index; Non-HDL = non–high-density lipoprotein cholesterol; RC = remnant cholesterol; AIP = atherogenic index of plasma; CRI-I = Castelli's risk index I; CRI-II = Castelli's risk index II.
